# Analysis of causes of death among brought-in-dead cases in a third-level Hospital in Lusaka, Republic of Zambia, using the tariff method 2.0 for verbal autopsy: a cross-sectional study

**DOI:** 10.1186/s12889-020-08575-y

**Published:** 2020-04-10

**Authors:** Yuta Yokobori, Jun Matsuura, Yasuo Sugiura, Charles Mutemba, Martin Nyahoda, Chomba Mwango, Lloyd Kazhumbula, Motoyuki Yuasa, Clarence Chiluba

**Affiliations:** 1 National Center for Global Health and Medicine (NCGM), 1-21-1, Toyama, Shinjuku-ku, Tokyo, Japan; 2 Ministry of Health (MoH), Zambia, Ndeke house, Lusaka, Zambia; 3 Adult Hospital, University Teaching Hospital, Ridgeway Nationalist Road, Lusaka, Zambia; 4 Department of National Registration, Passport & Citizenship, Ministry of Home Affairs, Cnr Dedani Kimathi & Independence roads, Lusaka, Zambia; 5 Department of Public Health, Graduate School of Medicine, Juntendo University, Hongo 2-1-1, Bunkyo-ku, Tokyo, Japan

**Keywords:** Civil registration and vital statistics, Death, Africa, Zambia, International classification of disease, Automated verbal autopsy, Verbal autopsy, SmartVA

## Abstract

**Background:**

Over one third of deaths in Zambian health facilities involve someone who has already died before arrival (i.e., Brough in Dead), and in most BiD cases, the CoD have not been fully analyzed. Therefore, this study was designed to evaluate the function of automated VA based on the Tariff Method 2.0 to identify the CoD among the BiD cases and the usefulness by comparing the data on the death notification form.

**Methods:**

The target site was one third-level hospital in the Republic of Zambia’s capital city. All BiD cases who reached the target health facility from January to August 2017 were included. The deceased’s closest relatives were interviewed using a structured VA questionnaire and the data were analyzed using the SmartVA to determine the CoD at the individual and population level. The CoD were compared with description on the death notification forms by using t-test and Cohen’s kappa coefficient.

**Results:**

One thousand three hundred seventy-eight and 209 cases were included for persons aged 13 years and older (Adult) and those aged 1 month to 13 years old (Child), respectively. The top CoD for Adults were infectious diseases followed by non-communicable diseases and that for Child were infectious diseases, followed by accidents. The proportion of cases with a determined CoD was significantly higher when using the SmartVA (75% for Adult and 67% for Child) than the death notification form (61%). A proportion (42.7% for Adult and 46% for Child) of the CoD-determined cases matched in both sources, with a low concordance rate for Adult (kappa coefficient = 0.1385) and a good for Child(kappa coefficient = 0.635).

**Conclusions:**

The CoD of the BiD cases were successfully analyzed using the SmartVA for the first time in Zambia. While there many erroneous descriptions on the death notification form, the SmartVA could determine the CoD among more BiD cases. Since the information on the death notification form is reflected in the national vital statistics, more accurate and complete CoD data are required. In order to strengthen the death registration system with accurate CoD, it will be useful to embed the SmartVA in Zambia’s health information system.

## Background

Information about deaths is essential for effective policy-making in order to address local demands in relation to various public health issues. The causes of death (CoD) information is especially significant for guiding health policy for the prioritization of health issues [1]. Ideally, all deaths should be registered and the CoD data should be obtained from accurate medical certification [2]. However, according to the World Health Statistics in 2015 [3], most low- and middle-income countries (LMIC) have a low death registration rate and inaccurate vital statistics [4]. In this context, strengthening death registration has attracted international attention as an important issue in the global public health field. The United Nations goal 16 of the sustainable development goals [5, 6] contains a target related to civil registration and vital statistics (CRVS) in order to promote human security.

The Republic of Zambia is one such country that is faced with substantial challenges to collecting accurate death information. According to the Central Statistics Office in Zambia, the death registration rate was 20% in 2016 [7]. One reason for the low registration rate is the large proportion of out-of-facility deaths. According to the Sample Vital Registration with Verbal Autopsy (SAVVY) which is standardized survey to investigate the CoDs of community death by using verbal autopsy, approximately 50% of the deceased died at home [8]. In addition, the health information system of the Zambian Ministry of Health in 2016 revealed that more than one third of death cases in health facilities died before their arrival, which are called brought-in-dead (BiD) cases. However, the CoD of these BiD cases have not yet been thoroughly analyzed. The background information of the BiD cases is one of the key factors for understanding the bottlenecks in the health system that obstruct the diseased from accessing the facilities before death. However, only a few publications have analyzed BiD cases in LMIC [9], including the African nations [10, 11].

In order to classify the CoD of these BiD cases, verbal autopsy (VA): a method of collecting information about symptoms and conditions for a deceased individual to determine his or her CoD, is now considered a realistic alternative to the medical certification of CoD [12]. The World Health Organization (WHO) has recommended using VA to strengthen the vital statistics regarding deaths to capture the CoD and trends in the national population [13]. While many countries [14–17] have incorporated VA into their official health information system, physician-based VA for all BiD cases may not be feasible in terms of the workload and costs. Therefore, automated VA is being developed to enable non-physician healthcare staff to make a reliable diagnosis of the CoD of BiD cases by using a computer program that can assign the most probable CoD by asking the deceased’s closest relatives the relevant questions.

Among several types of automated VA program, the Tariff Method 2.0 is one of the standardized tools that can be used to predict individual CoD by estimating the cause specific mortality fraction (CSMF) that is based on a comparison with the Population Health Metrics Research Consortium (PHMRC) gold standard database [18]. Previous studies have shown that the Tariff Method 2.0 can satisfactorily diagnose the CoD from the data obtained from the structured VA questionnaires in terms of its validity [18–23]. The WHO recommends the Tariff Method 2.0 as one of the effective automated VA tools [19, 20]. However, since none of the VA tools have been routinely used in Zambia, the CoD of most BiD cases have not been investigated in detail. Therefore, this study was designed to evaluate the function of automated VA based on the Tariff Method 2.0 to identify the CoD among the BiD cases who visited the main health facility in Zambia, and the usefulness by comparing the data with the written CoD information on the death notification form.

## Methods

### Research design

This research was designed as Cross-Sectional Study to analyze causes of death among BiD cases in a third level hospital in Lusaka, Republic of Zambia.

### Target site, population, and research period

The target site was a third-level hospital, the University Teaching Hospital (UTH), which is located in the capital of Zambia (Lusaka City). This city has a population of approximately 2.5 million. Since this health facility was the only third-level hospital in Lusaka that had a forensic doctor at the beginning of the research, most BiD cases in Lusaka were referred to the UTH. The target population was all BiD cases who reached the target health facility during the research period. BiD was defined as a death case who had already died before arrival at the hospital. The neonatal population aged 1 month and younger was excluded because neonatal death cases are usually transferred to a different ward in the UTH. The research period was for 4 months from May to August 2017. While the data should be collected for 1 year because of seasonal changes in the epidemiological profile, the limitation of resources for the research restricted the research period.

### Data collection

Once the BiD cases reached the BiD ward at the UTH, the interviewers employed for this research interviewed the deceased’s closest relatives using a paper-based questionnaire designed for the Tariff Method 2.0 for VA, which was developed by the WHO and Institute for Health Metrics and Evaluation (IHME); that is, the paper version of the PHMRC Shortened Questionnaire [24] which validity is discussed by Peter Serina et.al [18]. The researcher trained interviewers and made a 2-week pilot study before actual data collection. Then, the interviewers conducted the paper-based questionnaire by English and converted the data collected into an electronic format by using an application on an Android tablet called Open Data Kit (ODK) Collect [25]. The data were then stored in ODK Collect and transferred to the researchers’ personal computers via the internet by using a cloud-based data management program called ODK Aggregate. In addition, the interviewers collected and recorded what was written about the CoD on the death notification form issued for the same BiD cases in order to compare the CoD with the results of the automated VA. The death notification form is an official template used to record the backgrounds of the death cases in Zambia, such as the name, address, age, and CoD certified by medical doctors. The relatives are required to submit the death notification form to the civil registration office in order to obtain the death registration, which is necessary to gain the burial permission. Usually, there is insufficient information to determine the CoD on the death notification form of the BiD cases.

### Data analysis

The researchers extracted the data from ODK Aggregate as Comma Separated Value (CSV) files and uploaded them into the SmartVA program [25], which is a computer program developed by the IHME to assign individual CoD by using the Tariff Method 2.0, in order to identify the top 10 CoD of the adult and child BiD cases in the UTH. In order to automatically assign individual CoD, tariffs were created as CoD-specific normalized endorsement rates for each symptom reported in the PHMRC gold standard dataset. The tariff scores were calculated by summing all of the tariffs for the symptoms that were endorsed by the VA. Once the tariff scores were calculated for all of the samples in the dataset, they were compared to those whose true CoD was known from the PHMRC gold standard VA dataset. The CoD with the best tariff score, when compared to the gold standard VA, was then assigned using the Tariff Method 2.0’s 34 categories for persons aged 13 years and older and 21 categories for those aged 1 month to 13 years [26]. In this research, persons for the cases aged 13 years old and older are called as “Adult” and those aged 1 month to 13 years are called “Child”. The CoD were categorized using the Tariff Method 2.0, which corresponds to the codes of the International Classification of Diseases-10th revision (ICD-10). If a VA in the input data has tariff scores that are significantly lower than all of the tariff scores in the PHMRC gold standard dataset, the CoD is marked as “undetermined” for the individual estimates. These causes are redistributed to the CSMF based on country-specific cause fractions for the population-level estimates.

The CoD obtained using the SmartVA were compared with those on the death notification form in terms of the CoD-determination rate and concordance rate. In order to compare the CoD on the death notification form, the descriptions about the CoD on the death notification form were assigned to an ICD-10 code by the research team, using as many categories as those assigned by the SmartVA. The CoD were marked as undetermined if the death notification form described only symptoms, such as fever, pain, and coughing; unspecific conditions, such as natural death, sudden death, and witchcraft; or other unidentifiable words.

All variables were processed using the STATA version 14 (StataCorp., College Station, TX, USA) and Microsoft Excel 2013 (Microsoft, Redmond, WA, USA) software. The statistical differences were estimated using the independent-samples *t*-test. The concordance rate was estimated using Cohen’s kappa coefficient. For the VA, the SmartVA was utilized to estimate the CSMF by using the PHMRC Shortened Questionnaire and Tariff Method 2.0. The accepted level of significance is *p*-value< 0.05 and the concordance level by kappa coefficient is considered excellent if 1.0–0.8, good if 0.6–0.8, moderate if 0.4–0.6 and poor if 0.6 and below [27].

## Results

### The characteristics of the BiD cases

**Fig. 1:**
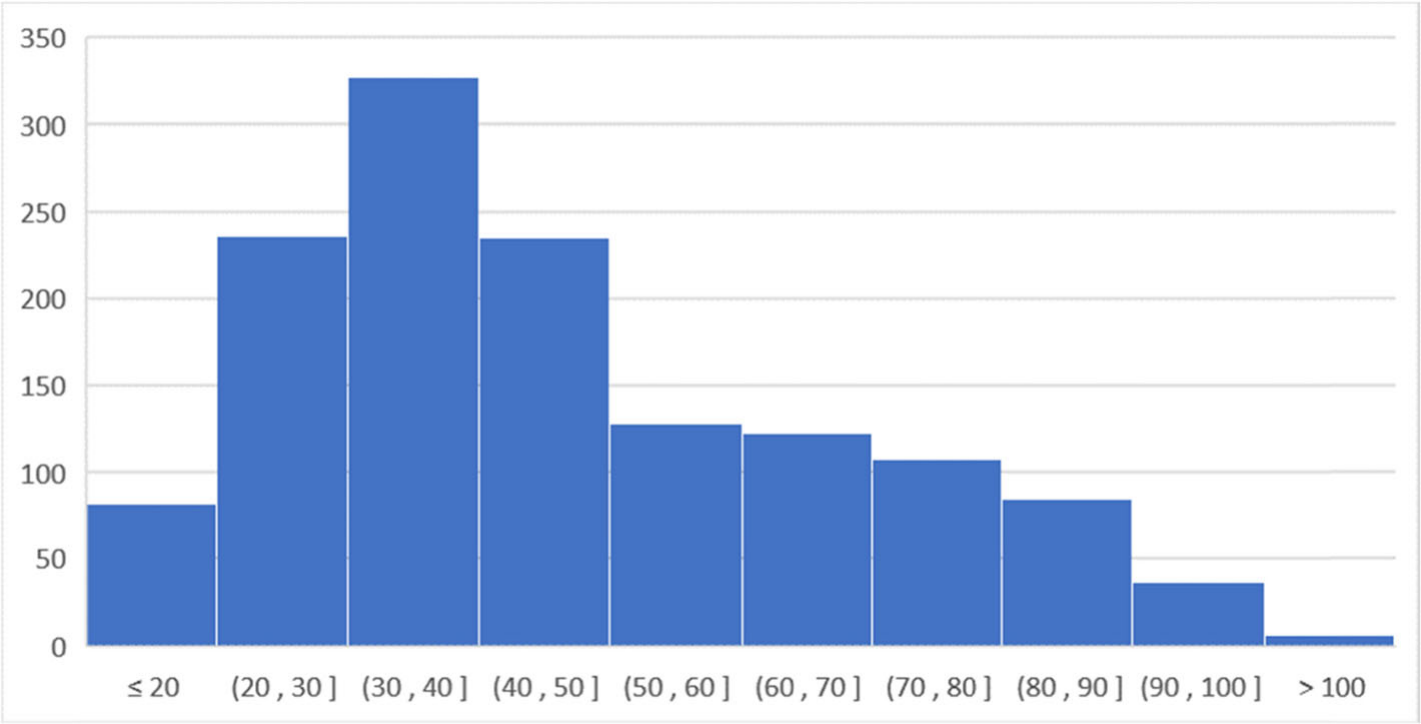
Age distribution of the adult brought-in-dead cases who were aged 13 years and older. The data were collected in the University Teaching Hospital during the research period

**Fig. 2:**
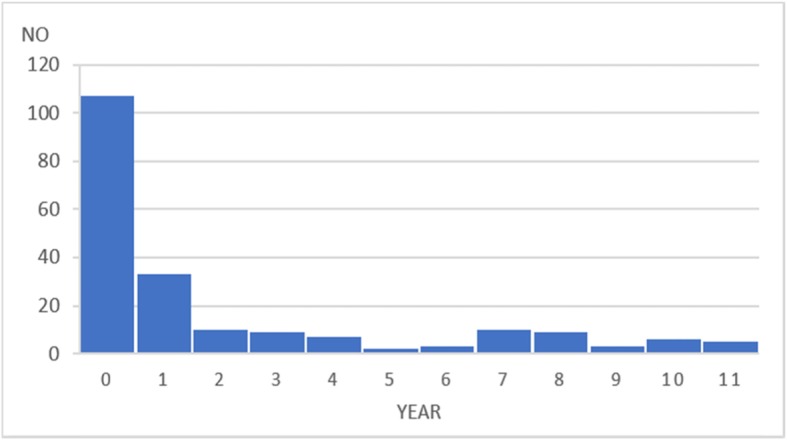
Age distribution of the child brought-in-dead cases who were aged 1 month to 13 years. The data were collected in the University Teaching Hospital during the research period

Table 1 describes the characteristics of the adult BiD cases aged 13 years and older and child BiD cases aged 1 month to 13 years in the UTH. The total number of cases was 1378 for adults and 209 for children during the 4-month research period from May to August 2017. There were 16 refusal cases in total. The most of reasons for refusals were respondents’ busyness to handle the funerals and emotional strain on the deceased. These refusal cases were simply excluded from the analysis since the number was small. Regarding the sex, 62.8% of adult cases and 44.9% of child cases were male. The median ages of the adults and children were 42.0 and 2.08 years old, respectively. Figures 1 and 2 present the age distribution according to the four age categories for the adult cases and by year for the child cases. Both figures show that there was not a normal distribution as the age of the adult cases had two peaks of distribution and the child BiD occurred more frequently in the younger ages. Most BiD cases originated from within Lusaka District.

**Table 1:** Characteristics of the brought-in-dead adult and child cases in the University Teaching Hospital

	Adult	Child
Total Number	1378	209
Number of Refusals	12	4
Male (%) (95% CI)	62.8 (60.2–65.4)	44.9 (38.0–51.7)
Age (median year: 25–75th percentile)	42.0 (34.0–65.0)	0.88 (0.42–3.0)
Origins of the Participants		
Within Lusaka	1299	190
Outside of Lusaka	44	5
Unknown Origin	23	10

### The CoD among the adult BiD cases aged 13 years and older

Table 2 describes the top 10 CoD among the adult BiD cases according to the SmartVA and death notification form. Among 1366 BiD cases in the UTH from May to August 2017, the top 10 CoD calculated by the SmartVA were, in order, acquired immunodeficiency syndrome (AIDS), stroke, tuberculosis (TB), suicide, diabetes mellitus (DM), other cardiovascular diseases (CVDs), pneumonia, epilepsy, acute myocardial infarction, and asthma. While two main infectious diseases each occupied one of the highest ranks of the CoD, there were also several non-communicable diseases (NCDs). However, there were several discrepancies with the death notification form. Firstly, AIDS was not found in the top CoD; instead, TB and malaria ranked higher. Secondly, diarrhea/dysentery was replaced with pneumonia, and cancers and CVDs were ranked higher than stroke, epilepsy, and asthma.

**Table 2:** Top 10 Causes of deaths among the brought-in-dead adult cases in the University Teaching Hospital during research period by SmartVA and Death Notification Form

SmartVA (*N* = 1366)				Death Notification Form (N = 1366)			
Rank	Cause of Death	No	%	Rank	Cause of Death	No	%
	Undetermined	344	25.2%		Undetermined	526	38.5%
1	HIV/AIDS	278	20.4%	1	TB	239	17.5%
2	Stroke	134	9.8%	2	Other CVDs	148	10.8%
3	TB	87	6.4%	3	Malaria	75	5.5%
4	Suicide	68	5.0%	4	Stroke	59	4.3%
5	DM	67	4.9%	5	Other Cancers	45	3.3%
6	Other CVDs	57	4.2%	6	Suicide	33	2.4%
7	Pneumonia	52	3.8%	7	Diarrhea/Dysentery	31	2.3%
8	Epilepsy	38	2.8%	8	Other NCDs	28	2.0%
9	IHD/AMI	26	1.9%	9	DM	25	1.8%
10	Asthma	25	1.8%	10	RTA	25	1.8%

NB: *TB* tuberculosis, *DM* diabetes mellitus, *CVDs* cardiovascular diseases, *AMI* acute myocardial infarction, *IHD* ischemic heart disease, *NCDs* non-communicable diseases, *RTA* road traffic accident

If a case’s tariff score was not significantly higher for a specific health condition than for other conditions, the SmartVA labeled it as an undetermined case. In addition, in the death notification form, some descriptions, such as fever, pain, or natural death, could not be properly coded. Table 3 shows the number of determined CoD of the BiD cases according to the SmartVA and death notification form. The proportion of BiD cases whose CoD were determined was 61% for the death notification form and 75% for the SmartVA. The results of the statistical analysis showed that the SmartVA significantly improved the determination rate of the CoD compared with the death notification form. Table 4 presents the number of BiD cases whose CoD were not determined by the death notification form and SmartVA. The proportion of BiD cases with a determined CoD according to both methods was 686 (50.2%). Regarding these cases, Table 4 shows that 43.3% of the CoD matched between the death notification form and SmartVA, and the concordance rate was low in term of the kappa coefficient (0.083 95% confidence interval [CI]: 0.069–0.089).

**Table 3:** Number of the brought-in-dead adult cases with determined causes of death by both of Death Notification Form and SmartVA in the University Teaching Hospital

	No. of Cases With Determined CoDs	Mean	95% CI	*P*-value
Death Notification Form	840	0.61	0.589–0.641	
SmartVA	1021	0.75	0.724–0.771	< 0.05

**Table 4:** The number of undetermined cases among the brought-in-dead adult cases and the concordance rate of CoDs among the cases with determined CoDs by both of Death Notification Form and SmartVA in the University Teaching Hospital

		Number	Percentage
Both Undetermined		191	14.0%
Undetermined only by Death Notification Form		335	24.5%
Undetermined only by SmartVA		154	11.3%
Both Determined		686	50.2%
Matched CoDs between both methods		297	43.3%
Unmatched CoDs between both methods		389	56.7%
Kappa coefficient		0.083 (95% CI: 0.069–0.086)	

NB: *CoDs* cause of deaths

Additional file 1 shows the distribution of the CoD among the adult BiD cases according to the four age categories: 13–19 years, 20–44 years, 45–59 years, and 60 years and above. The proportion of undetermined cases was largest among people aged 13–19 years according to the death notification form (51.3%) and smallest among those aged 45–59 years according to the SmartVA (19.9%). Generally speaking, there was less malaria and more AIDS in the SmartVA group, compared with the death notification form, in the distribution of the CoD and the older the BiD cases became, the higher the number of NCDs there were in the top CoD. In addition, there were further differences between the CoD according to the SmartVA and death notification form. For example, the SmartVA identified more suicide cases among the younger population and more neurological conditions (stroke or epilepsy) and DM cases in all generations. Indeed, the concordance rates between the SmartVA and death notification form were weak among all of the age categories in terms of the kappa coefficient, whereas this coefficient was higher in the middle-aged categories (20–59 years) than in people aged 13–19 years and 60 years and above.

### The CoD among the child BiD cases aged 1 month to 13 years

Table 5 describes the top 10 CoD among the adult BiD cases according to the SmartVA and death notification form. Among 205 BiD cases in the UTH from May to August 2017, the top 10 CoD calculated by the SmartVA were, in order, pneumonia, diarrhea/dysentery, human immunodeficiency virus (HIV)/AIDS, malaria, fires, drowning, other CVDs, meningitis, other defined CoD, and road traffic accident (RTA). Furthermore, infectious diseases, such as pneumonia, diarrhea/dysentery, and HIV/AIDS occupied the highest ranks of the CoD, followed by accidents, such as fire, drowning, and RTA. While the distribution of the CoD according to the death notification form was similar to that of the SmartVA, there were fewer HIV/AIDS cases on the death notification form and more epilepsy cases than meningitis cases. Table 6 shows how many of the CoD of the BiD cases were determined by the SmartVA and death notification form. The proportion of the BiD cases whose CoD were determined was 46% for the death notification form and 67% for the SmartVA. The statistical analysis indicated that the SmartVA significantly improved the determination rate of the CoD compared with the death notification form. Table 7 presents the number of BiD cases whose CoD were not determined by the death notification form and SmartVA. The proportion of BiD cases with determined CoD for both methods was 77 (37.6%). Regarding these cases, Table 7 shows that 68.8% of the CoD were matched between the death notification form and SmartVA and the concordance rate was substantial in terms of the kappa coefficient (0.635, 95% CI: 0.548–0.740).

**Table 5:** Top 10 Causes of deaths among the brought-in-dead child cases in the University Teaching Hospital during research period by SmartVA and Death Notification Form

SmartVA (*N* = 205)				Death Notification Form (N = 205)			
Rank	Cause of Death	No.	%	Rank	Cause of Death	No.	%
	Undetermined	68	33.2%		Undetermined	111	54.1%
1	Pneumonia	36	17.6%	1	Diarrhea/Dysentery	18	8.8%
2	Diarrhea/Dysentery	32	15.6%	2	Other Defined CoD	14	6.8%
3	HIV/AIDS	25	12.2%	3	Pneumonia	10	4.9%
4	Malaria	5	2.4%	4	Malaria	9	4.4%
5	Fires	5	2.4%	5	RTA	7	3.4%
6	Drowning	5	2.4%	6	Drowning	7	3.4%
7	Other CVDs	5	2.4%	7	Fires	6	2.9%
8	Meningitis	4	2.0%	8	Other CVDs	6	2.9%
9	Other Defined CoD	4	2.0%	9	Epilepsy	5	2.4%
10	RTA	4	2.0%	10	Other Cancers	5	2.4%

NB: *CVDs* cardiovascular diseases, *RTA* road traffic accident

**Table 6:** Number of the brought-in-dead child cases with determined causes of death by both of Death Notification Form and SmartVA in the University Teaching Hospital

	No. of Cases With Determined CoDs	Mean	95% CI	*P*-value
Death Notification Form	94	0.46	0.390–0.527	
SmartVA	137	0.67	0.603–0.733	< 0.05

**Table 7:** The number of undetermined cases among the brought-in-dead child cases and the concordance rate of CoDs among the cases with determined CoDs by both of Death Notification Form and SmartVA in the University Teaching Hospital

	Number	Percentage
Both Undetermined	51	24.9%
Undetermined only by Death Notification Form	17	33.2%
Undetermined only by SmartVA	60	62.4%
Both Determined	77	37.6%
Matched CoDs between both methods	53	68.8%
Unmatched CoDs between both methods	24	31.2%
Kappa coefficient	0.635 (95% CI: 0.548–0.740)	

NB: *CoDs* cause of deaths

## Discussion

This research analyzed the CoD of 1366 adult and 205 child BiD cases who were brought into a third-level health facility in the capital city of Zambia by using an automated VA tool, called SmartVA, for the first time. The results indicate that the top CoD among the adult cases were infectious diseases, including AIDS, TB, and malaria, followed by NCDs, such as stroke, CVDs, and DM, and that those among the child cases were mainly infectious diseases, such as pneumonia, diarrhea, and malaria, followed by accidents. These trends are similar to the distribution of the CoD data of the Global Burden of Disease [28].

The comparison of the CoD between the SmartVA and death notification form showed that there are discrepancies in the distributions of the CoD among the adult cases; that is, there were fewer HIV/AIDS cases and more malaria cases in the death notification form. There were also fewer HIV/AIDS cases among the child cases in the death notification form. One possible reason is that the coders misclassified the CoD if a death case had two or more health conditions that lead to death. The WHO’s instruction manual for ICD-10 coding [29] states in page 35, “When more than one condition is entered on the certificate, select the condition entered alone on the lowest used line of underlying causes of death only if it could have given rise to all the conditions entered above it.” However, the coders for the death notification form may have reported the more immediate health conditions leading to death as the CoD. In that case, HIV/AIDS would be less likely to be selected as the final CoD.

In addition, there were more cases with an undetermined CoD on the death notification form compared with the SmartVA. This may be due to the healthcare staff’s lack of knowledge about recording CoD. The symptoms, such as pain or fever, should not be noted as the CoD and unspecified health conditions, such as natural death or sudden death, should also be avoided. Thus, creating a standardized procedure for recording the CoD might be necessary.

Furthermore, the concordance rate between the SmartVA and death notification form was low especially among the adult BiD cases. This trend was the same among the different age categories, although the concordance rate was slightly higher in the middle-aged category (20–59 years) than in people aged 13–19 years and 60 years and above. One of the main reasons behind this discrepancy could be the larger number of undetermined cases and misclassification on the death notification form. Especially, the small number of AIDS cases as the CoD on the death notification form is not consistent with what is already known about this disease burden in Zambia [26]. Since these CoD on the death notification form are reflected in the national vital statistics data, it is a problem that the actual data reported to the Civil Registration Office are skewed. The establishment of a system to improve the accuracy of the CoD among the BiD cases is necessary to capture the accurate vital statistics through the CRVS in a timely manner. Automated VA using the SmartVA is one option for strengthening the vital statistics since it can improve the quality of the determination of the individual CoD and can be used to estimate the population level of the CoD from all samples.

There are several limitations in this research. Firstly, the research period in the UTH was only 4 months because of financial and human resource constraints. However, seasonal fluctuation needs to be considered for some health conditions, such as malaria, diarrhea, and probably some NCDs. Therefore, the results of this research cannot be completely generalized as annual data. However, since most health conditions do not vary by month, according to the existing health information system, these conditions that do not exhibit seasonal differences can be generalized as annual data. In addition, regarding generalizability, another limitation is that this research included the death cases brought to hospital, but not the death cases at home. In order to interpret burden of diseases in Lusaka, further research should be required in the future.

Secondly, there were several BiD cases in the UTH that were not included in this research. For example, some of children BiD cases were not covered because the UTH has another specific ward for children cases and some of these BiD cases were directly carried to the children wards. Such BiD cases in the children wards were unfortunately not well recorded. Furthermore, neonatal BiD cases were not captured because most of them were dealt with as hospital deaths once they were delivered to the neonatal intensive care unit. However, since most of the child BiD cases that were older than 1 month were directly carried to the BiD ward, this research did not just cover neonatal BiD cases. It is desirable to conduct another research study to investigate neonatal BiD cases in the UTH.

Thirdly, there was a substantial number of BiD cases with an undetermined CoD, even when using the SmartVA, although its determination rate was significantly better than that of the death notification form. When the SmartVA was used to analyze the top 10 CoD among the same research samples by calculating the CSMF as the population level, there were discrepancies in the distribution compared to the results of the individual analysis in terms of there being more malaria and more other NCDs (Additional file 2). These differences may derive from the large number of undetermined cases, which account for approximately one fourth of the samples. In order to make the results reliable, the computer algorithm that determines the CoD should be improved to reduce the number of undetermined BiD cases in the future.

Lastly, the validity of the Tariff Method 2.0 for identifying the CoD by using the SmartVA should be considered more. According to Serina et al. [10], the chance-corrected concordance used to assess the extent to which the Tariff Method 2.0 correctly predicts an individual’s CoD and the CMSF’s accuracy for measuring the performance at the population level, when applied to the shortened version of the PHMRC Questionnaire, were 50.0 and 76.6% respectively for the adult population. However, since this validity was investigated by comparing it with the gold standard dataset [19], the applicability to the Zambian setting should be scrutinized by investigating the actual data. In addition, new automated VA tools have been developed, such as the InterVA-5 [30], Naïve Bayes Classifier [31, 32], SilicoVA [33], and so on. We also need to investigate the functionality, feasibility, and validity of these new tools. In the future, a validation study that compares the CoD by using an automated VA tool with those determined by an actual full autopsy is required to estimate the true burden of diseases in Zambia.

## Conclusions

By using an automated VA tool (i.e., the SmartVA that is based on the Tariff Method 2.0), the CoD of the BiD cases in the UTH have been successfully analyzed for the first time in Zambia. The SmartVA can determine the CoD of more BiD cases, when compared to the death notification form. In addition, the distribution of the CoD was inconsistent because there was a large number of erroneous descriptions of the CoD on the death notification form. Since these CoD on the death notification form are reflected in the national vital statistics data, it is a problem that the actual data reported to the Civil Registration Office are skewed. In order to strengthen the death registration system with accurate CoD, it will be useful to embed the automated VA into Zambia’s health information system. However, further investigation into the validity of the CoD identified by the automated VA should be considered in the future.

## Supplementary information

**Additional file 1.** Top 10 causes of death among adult brought-in-dead cases by age-categories during research period by SmartVA and Death Notification Form.

**Additional file 2.** Top 10 causes of death according to the individual level and population level analysis.

## Data Availability

The datasets generated and/or analyzed during the current study are not publicly available due to an ethical restriction (patient confidentiality) but are available from the corresponding author on reasonable request.

## Abbreviations

AIDS: Acquired immunodeficiency syndrome
AMI: Acute myocardial infarction
BiD: Brought in dead
CI: Confidence interval
CoD: Causes of death
CRVS: Civil registration and vital statistics
CSMF: Cause specific mortality fraction
CVDs: Cardiovascular diseases
DM: Diabetes mellitus
HIV: Human immunodeficiency virus
ICD-10: International Classification of Diseases-10th revision
IHD: Ischemic heart disease
IHME: Institute for Health Metrics and Evaluation
LMIC: Low- and middle-income countries
NCDs: Non-communicable diseases
ODK: Open data kit
PHMRC: Population Health Metrics Research Consortium
RTA: Road traffic accident
TB: Tuberculosis
UTH: University Teaching Hospital
VA: Verbal autopsy
WHO: World Health Organization
