# Supplementary material for: Analysis of causes of death among brought-in-dead cases in a third-level Hospital in Lusaka, Republic of Zambia, using the tariff method 2.0 for verbal autopsy: a cross-sectional study
Source: BMC Public Health. 2020 Apr 10;20:473. doi: 10.1186/s12889-020-08575-y (PMC7147005; doi:10.1186/s12889-020-08575-y)
Supplement: Supplementary file 1 — Additional file 1. Top 10 causes of death among adult brought-in-dead cases by age-categories during research period by SmartVA and Death Notification Form. [file 12889_2020_8575_MOESM1_ESM.docx]

| Additional File 1:Top 10 causes of death among adult brought-in-dead cases by age-categories during research period by SmartVA and Death Notification Form | | | | | | | | | | | | | | | | |
| --- | --- | --- | --- | --- | --- | --- | --- | --- | --- | --- | --- | --- | --- | --- | --- | --- |
| Age | 13-19 year | | | | 20-44 year | | | | 45-59 year | | | | 60 year and above | | | |
| Method | SmartVA | | DNF | | SmartVA | | DNF | | SmartVA | | DNF | | SmartVA | | DNF | |
| Total | 78 | | 78 | | 743 | | 743 | | 277 | | 277 | | 410 | | 410 | |
| UD | 23 (29.5%) | | 40(51.3%) | | 194(26.1%) | | 298(40.1%) | | 55(19.9%) | | 102(36.8%) | | 104(25.4%) | | 142(34.6%) | |
| Rank | CoD | No | CoD | No | CoD | No | CoD | No | CoD | No. | CoD | No. | CoD | No. | CoD | No. |
| 1 | Suicide | 8 | Malaria | 9 | AIDS | 204 | TB | 170 | AIDS | 68 | TB | 49 | Stroke | 83 | Other CVD | 99 |
| 2 | AIDS | 7 | TB | 5 | TB | 54 | Malaria | 47 | Stroke | 24 | Other CVD | 40 | Diabetes | 30 | Stroke | 46 |
| 3 | Stroke | 6 | Poisoning | 5 | Suicide | 54 | Other CVD | 27 | TB | 20 | Malaria | 16 | Other CVD | 26 | TB | 28 |
| 4 | Epilepsy | 5 | Suicide | 5 | Stroke | 35 | Suicide | 26 | Diabetes | 20 | Other Cancer | 14 | TB | 21 | Other Cancer | 22 |
| 5 | Diarrhea | 4 | AIDS | 2 | Pneumonia | 30 | Diarrhea | 25 | Other CVD | 14 | Stroke | 13 | Pneumonia | 21 | Diabetes | 15 |
| 6 | TB | 3 | Other NCD | 2 | Epilepsy | 24 | Other NCD | 21 | AMI | 12 | RTA | 7 | Asthma | 20 | Asthma | 10 |
| 7 | Maternal | 3 | Drowning | 2 | Diabetes | 19 | Poisoning | 21 | Epilepsy | 8 | Diabetes | 6 | AIDS | 16 | Renal Failure | 8 |
| 8 | Renal Failure | 3 | Diarrhea | 1 | Other CVD | 18 | RTA | 15 | Other injuries | 7 | Renal Failure | 6 | AMI | 13 | Malaria | 7 |
| 9 | Poisoning | 3 | Maternal | 1 | RTA | 15 | Homicide | 12 | RTA | 6 | Poisoning | 4 | Other NCD | 12 | RTA | 6 |
| 10 | Diabetes | 3 | Epilepsy | 1 | Poisoning | 13 | Other Cancer | 10 | Diarrhea | 5 | Other Infection | 3 | Cervical Cancer | 9 | Other NCD | 5 |
|  |  |  | Homicide | 1 |  |  | Pneumonia | 10 | Pneumonia | 5 | Other NCD | 3 |  |  |  |  |
|  |  |  | RTA | 1 |  |  |  |  | Renal Failure | 5 |  |  |  |  |  |  |
|  |  |  |  |  |  |  |  |  | Suicide | 5 |  |  |  |  |  |  |
| kapper (95%CI) | 0.051(-0.007-0.124) | | | | 0.092(0.082-0.103) | | | | 0.083(0.073-0.089) | | | | 0.023(0.016-0.027) | | | |
| NB: CoD: Cause of Death, DNF: Death Notification Form, UD: Undetermined, CVD: Cardiovascular Disease, NCD: Non-communicable Disease, RTA: Road Traffic Accident | | | | | | | | | | | | | | | | |
