# Supplementary material for: Analysis of causes of death among brought-in-dead cases in a third-level Hospital in Lusaka, Republic of Zambia, using the tariff method 2.0 for verbal autopsy: a cross-sectional study
Source: BMC Public Health. 2020 Apr 10;20:473. doi: 10.1186/s12889-020-08575-y (PMC7147005; doi:10.1186/s12889-020-08575-y)
Supplement: Supplementary file 2 — Additional file 2. Top 10 causes of death according to the individual level and population level analysis. [file 12889_2020_8575_MOESM2_ESM.docx]

| Additional File 2: Top 10 causes of death according to the individual level and population level analysis among the brough-in-dead adult cases. | | | | |
| --- | --- | --- | --- | --- |
|  | SmartVA (population level) | | SmartVA (individual level) | |
| Rank | CoD | CSMF | CoD | No. |
| 1 | HIV/AIDS | 0.260 | HIV/AIDS | 278 |
| 2 | Stroke | 0.109 | Stroke | 134 |
| 3 | TB | 0.077 | TB | 87 |
| 4 | Other NCDs | 0.061 | Suicide | 68 |
| 5 | Malaria | 0.054 | DM | 67 |
| 6 | DM | 0.053 | Other CVDs | 57 |
| 7 | Pneumonia | 0.052 | Pneumonia | 52 |
| 8 | Other CVDs | 0.050 | Epilepsy | 38 |
| 9 | Suicide | 0.050 | IHD/AMI | 26 |
| 10 | Epilepsy | 0.028 | Asthma | 25 |
| NB: Cases brought into the University Teaching Hospital and according to the SmartVA. CoD: causes of death, CSMF: cause specific mortality fraction, TB: tuberculosis, NCDs: non-communicable diseases, DM: diabetes mellitus, CVDs: cardiovascular diseases, AMI: acute myocardial infarction, IHD: Ischemic Heart Disease. | | | | |
